# The Valorization of Spent Coffee Ground Extract as a Prospective Insecticidal Agent against Some Main Key Pests of *Phaseolus vulgaris* in the Laboratory and Field

**DOI:** 10.3390/plants11091124

**Published:** 2022-04-21

**Authors:** Hany Hussein, Waleed Abouamer, Hatem Ali, Manal Elkhadragy, Hany Yehia, Amr Farouk

**Affiliations:** 1 Pests and Plant Protection Department, Institute of Agricultural and Biological Research, National Research Centre, Cairo 12622, Egypt; hhussein@hotmail.com; 2 Plant Protection Department, Faculty of Agriculture, Al-Azhar University, Cairo 12622, Egypt; wabouamer@azhar.edu.eg; 3 Food Technology Department, National Research Center, Cairo 12622, Egypt; 4 Department of Biology, College of Science, Princess Nourah Bint Abdulrahman University, P.O. Box 84428, Riyadh 11671, Saudi Arabia; mfelkhadragy@pnu.edu.sa; 5 Department of Food Science and Nutrition, College of Food and Agriculture Science, King Saud University, Riyadh 11451, Saudi Arabia; hanyehia@ksu.edu.sa; 6 Department of Food Science and Nutrition, Faculty of Home Economics, Helwan University, Cairo P.O. Box 11611, Egypt; 7 Flavour and Aroma Chemistry Department, National Research Centre, Cairo 12622, Egypt

**Keywords:** spent coffee grounds, bioinsecticide, HPLC, *Phaseolus vulgaris*, in silico studies

## Abstract

The exploitation of massive amounts of food and agro-waste represents a severe social, economic, and environmental issue. Under the growing demand for food products that are free of toxic synthetic insecticides, a methanolic extract of spent coffee grounds (SCGs), which represent the main byproduct of coffee production, was applied in the current study as a bioinsecticide against the main pests of the green bean: *Spodoptera littoralis*, *Agrotis ipsilon, Bemisia tabaci*, *Empoasca fabae*, and *Aphis craccivora*. A deterrent assay, contact bioassay, and lethal concentration analysis were performed to reveal the repellent, antifeedant, and oviposition deterrent effects. Parallel to the above-mentioned bioassays, the phytochemical composition of the methanolic SCG extract was investigated via a high-performance liquid chromatography (HPLC) analysis. Fourteen phenolic acids and five flavonoids, in addition to caffeine (alkaloid), were identified in the extract. Cinnamic, rosmarinic, and gallic acids were the predominant phenolics, while apigenin-7-glucoside was the main flavonoid, followed by naringin, catechin, and epicatechin. The extract of SCGs showed an insecticidal effect, with a mortality between 27.5 and 76% compared to the control (7.4%) and based on the concentration of the extract used. In the same trend, the oviposition efficiency revealed different batches of laid eggs (0.67, 2.33, 7.33, and 8.67 batches/jar) for 100, 50, and 25% of the SCG extract and the control. Finally, the major components of the SCG extract were docked into the insecticide acetylcholinesterase enzyme to explore their potential for inhibition, where apigenin-7-glucoside showed a higher binding affinity, followed by catechin, compared to the control (lannate). The obtained findings could be a starting point for developing novel bioinsecticides from SCGs.

## 1. Introduction

Many insects have greatly affected food production, human health, and, consequently, the global economy. However, the inappropriate use of synthetic insecticides to control such insects is related to the development of resistance in pests, human diseases, and contamination of both food and the environment [1]. Therefore, natural products with insecticidal activity constitute a vital alternative that could be described as safe for humans and the environment. Plants are a rich source of bioactive metabolites, such as phenolic acids, which are well known for their antioxidant, anticancer, and antimicrobial activities [2]. Previous studies showed that phenolic *J. regia* L. extract showed inhibition in fecundity and development of the grain aphid (*Sitobion avenae* L.) using lab bioassays [3]. In the same context, Czerniewicz et al. [4] indicated that phenolic acids from *H. perforatum* L., *J. regia* L., *M. piperita* L., and *S. nigra* L. negatively affected the development, deterrent, and aphidicidal activity of the peach-potato aphid, *Myzus persicae* Sulz., and the bird cherry-oat aphid, *Rhopalosiphum padi* L.

Agricultural and food-industrial solid wastes are accumulated in a vast quantity, represent a global problem for health and the environment, and could be a source for bioactive phenolics. So, the exploitation of these wastes as raw materials for the bio-economy became a target for food sustainability policy [5]. For instance, worldwide, more than 6.5 million tons of spent coffee grounds (SCGs) were obtained from roasted coffee beans during instant coffee production in 2020/2021 [6]. SCGs showed concerning alkaloids and phenolics with superior antioxidant and anti-inflammatory activities [7,8,9]. To our knowledge, coffee grounds and their waste were used as repellents for the females of the cockroach and gravid *Aedes albopictus* [10,11]; however, nothing was reported concerning the insecticidal activity of SCG extracts.

Common bean (*Phaseolus vulgaris*) is a mean grain legume crop that is present worldwide, and it is third in importance after the soybean and peanut. It is an important crop that is cultivated in Egypt, where the domestic production of this crop was 144 thousand tons in 2018. However, the expectation of a decrease in production to 16 thousand tons by 2025 may lead to an increase in beans imported from 410 thousand tons in 2018 to 500 thousand tons in 2025 [12]. In this crop, losses caused by insect pests alone have been estimated to be from 35% to 100% annually [13]. Insect pests, including aphids, caterpillars, leafhoppers, and whiteflies, are responsible for the excessive loss percentages of this crop. Several pests, such as lepidopteran and sucking pests, prefer to attack *P. vulgaris* plants [14].

This study investigates the insecticidal efficacy and the repellent effect of a methanolic SCG extract against five distractive pests of the *P. vulgaris* crop: the aphid, *Aphis craccivora*; leafhopper, *Empoasca fabae*; whiteflies, *Bemisia tabaci*; cotton leafworm, *Sopdoptera littoralis*; cutworm, *Agrotis ipsilon*. The toxic effects and deterrence were assessed with a lethal concentration assay, deterrent assay, and contact bioassay analysis. Following the screening and identification of the methanol SCG extract using HPLC, molecular docking and dynamic simulation were performed to evaluate compounds with potential insecticidal activity by predicting the binding modes of the major components of the extract with insect acetylcholinesterase (AChE), which controls synaptic transmission and is essential for neurotransmitter hydrolysis (acetylcholine). The valorization of SCGs as a novel environmentally friendly insecticide seems to be a business opportunity for high-value-added products, which can be established as an approach enclosed in the general idea of sustainability.

## 2. Results and Discussion

### 2.1. Characterization of Phenolic Acids, Flavonoids, and Caffeine Using HPLC

**Figure 1:**
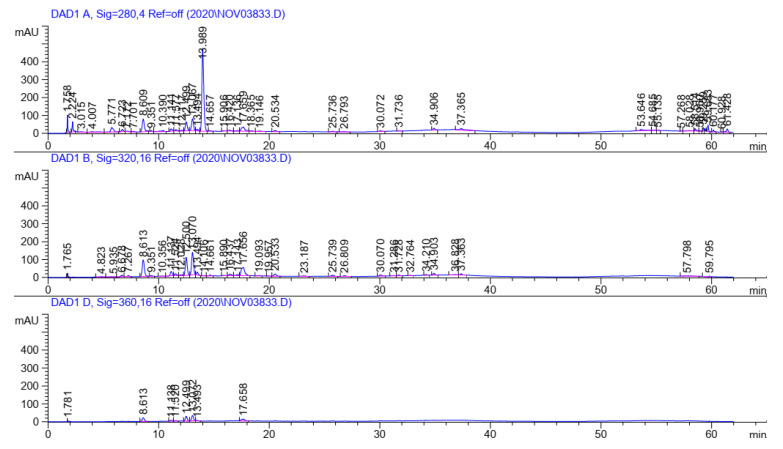
HPLC chromatograms of the methanolic SCG extract at 280, 320, and 360 nm.

Based on the HPLC analysis, fourteen phenolic acids and five flavonoids, in addition to caffeine (alkaloid), were identified in the methanolic SCG extract (Table 1, Figure 1). Cinnamic (979.38 µg/g), R-(+)-rosmarinic (163.10 µg/g), and gallic (32.51 µg/g) acids were the predominant phenolic acids, followed by sinapic (10.10 µg/g), chlorogenic (8.74 µg/g), salicylic (7.61 µg/g), and caffeic acids (6.41 µg/g) (Table 1). Many researchers have investigated the polyphenolic constituents of coffee waste; however, the yields and types of bioactive compounds extracted from SCGs depend on many factors, such as the coffee species, instant coffee production technique and efficiency, storage conditions, and the extraction method, which is based on polar solvents. For example, increasing the intensity of coffee roasting from medium to dark reduced phenolic acids such as chlorogenic acid due to isomerization, in addition to gallic, *p*-coumaric, and ferulic acids; ellagic acid was also increased without affecting the caffeic acid and ruten contents [9]. Andrade et al. [15] extracted phenolic compounds from SCGs and coffee husks using different techniques, such as ultrasonic, Soxhlet, and SFE techniques based on ethanol, ethyl acetate, and CO2 solvents. They identified chlorogenic acid as more abundant than the others in all extracts. Meanwhile, gallic, p-hydroxybenzoic, protocatechuic, vanillic, and tannic acid were also detected in lower concentrations. In contrast, Okur et al. [16] identified both chlorogenic and caffeic acids as minor components, which agrees with our findings. The focus on chlorogenic acid is due to its potential health benefits, as described in previous studies, including its free-radical-scavenging capacity and anti-inflammatory, antidiabetic, and anticancer effects [17].

Cinnamic acid, which is predominant in the present study, in agreement with Vamanu et al. [18], has generally been reported to be a phenolic constituent in SCGs in previous research, but in much lower concentrations with respect to the extraction technique [19]. As explained above, the variations might be due to many reasons, including the solvent, solvent/sample ratio, extraction time, extraction method, and temperature. To our knowledge, rosmarinic acid, an ester of caffeic acid reported in *Coffea arabica* L. [20], was newly identified in the present study and has never been mentioned before in SCGs. Compared to ethanol and methanol, water was reported as the most efficient solvent for the extraction of gallic acid; therefore, it was not always detected in SCGs [21]. However, gallic acid was identified in the current study with a median concentration, which might have been due to the detection and quantification sensitivity of the analysis [19]. On the other hand, coumaric (0.16 µg/g), gentisic (0.26 µg/g), and ferulic (0.37 µg/g) acids were the lowest compared to the identified constituents, which is in agreement with Angeloni et al. [22] (Table 1).

Apigenin-7-glucoside was the main flavonoid identified in the methanolic SCG extract, followed by naringin, catechin, epicatechin, and chrysin (Table 1). To our knowledge, only four flavonoids have been identified in SCGs by using ethanol and a microwave extraction technique: epicatechin, catechin, rutin, and quercetin; however, nothing was reported about apigenin-7-glucoside or naringin [9]. Generally, both apigenin-7-glucoside and naringin were present in green coffee beans, with an increase in their concentrations upon roasting using either microwave or oven techniques [23]. It is well known that flavonoids serve many beneficial health functions and elicit protective effects, including anti-inflammatory, antioxidant, antiviral, and anti-carcinogenic effects [9].

Caffeine is one of the essential bioactive components found in SCGs in a remarkable concentration (Table 1), consistently with Kovalcik et al. [7]. It is a methylxanthine alkaloid that shows deleterious effects on the nervous system, on the sensitization of DNA to damage, on the delayed entry of cells into mitosis, and on other aspects of cell division in the development of organisms, fertility, and chromatin structure, to mention but a few [24].

### 2.2. Oviposition Deterrent Activities of SCG Extract against the Female Moth Spodoptera littoralis

**Figure 2:**
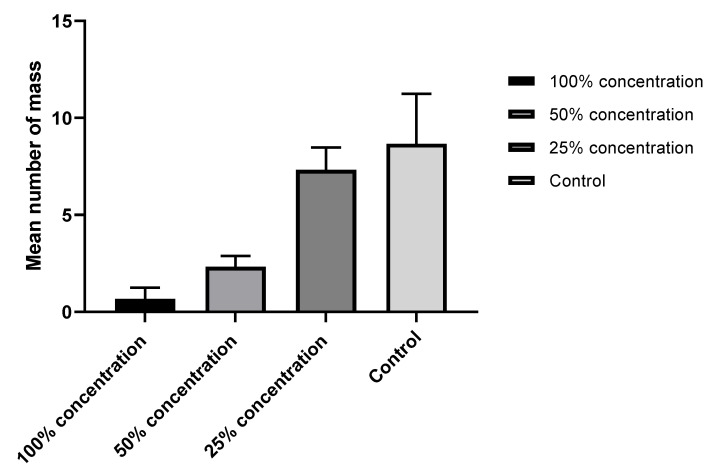
Effect of the SCG extract on the oviposition of *Spodoptera littoralis* females.

Daily inspection showed that eggs laid by females in jars treated with the maximum SCG extract (100%) were rarely observed (0.67 ± S.D 0.58 batch/jar). Although the mean number of eggs was minimal, females put their eggs on the inner wall of the jars, not on the treated leaves; on the other hand, females in non-treated jars laid their eggs entirely on the leaves. Meanwhile, the mean numbers of egg batches laid in each treatment ± S.D. were 2.33 ± 0.56, 7.33 ± 1.15, and 8.67 ± 2.57 in the treatments 50 and 25 and the control, respectively (Figure 2). The statistical analysis of the data showed extremely significant differences within concentrations, where the *p*-value was <0.0001. The results of the present investigation agree with those of Borges et al. [25], who showed the oviposition deterrent and larvicidal activities of the phenolic extracts of *T. avellanedae* against third-instar larvae of *Aedes aegypti*. In the same context, Kovanci [26] revealed the feeding and oviposition deterrent activities of microencapsulated cardamom oleoresin and eucalyptol against *Cydia pomonella*, while Basukriadi and Wilkins [27] studied the effect of yam bean seed extract and coumarin, which partially deterred the moth *Plutella xylostella*.

### 2.3. Insecticidal Effect of the SCG Extract on the Percentage of Mortality of Spodoptera littoralis

**Figure 3:**
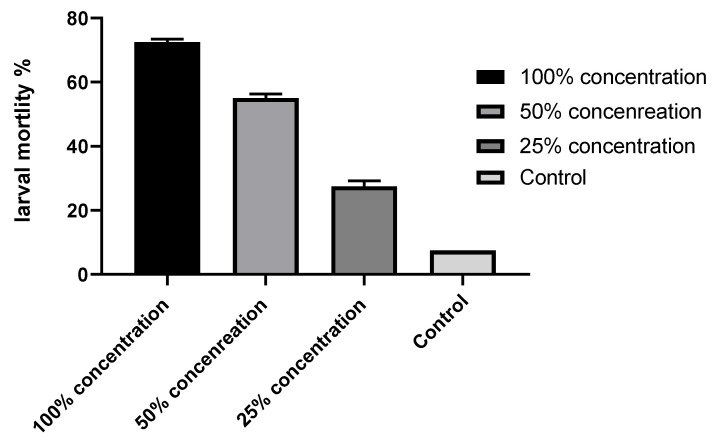
Percentage of mortality in *Spodoptera littoralis* larvae fed on leaves treated with the SCG extract.

The results showed that the SCG extract (100%) caused the mortality of 76% of larvae after four days of treatment. However, with a 50% extract concentration, the mortality reached 54.5%, and the lowest extract concentration, 25%, resulted in 27.5% mortality compared to the control, with 7.4% mortality in larvae (Figure 3). The statistical analysis with ANOVA showed significant differences between the treatments and the control, where the *p*-values were 0.034, 0.0301, and <0.001 for the concentrations of 25, 50, and 100% compared to the control. The main remark from our observations during this test was that there was no consumption of treated leaves, unlike in the control dishes, where the larvae fed on and consumed many parts of the leaves. This observation reveals that the SCG extract can act as a repellent or antifeedant agent against the larvae of *Spodoptera littoralis*, which died without feeding on treated leaves.

These results agreed with those of Pavela [28], who showed the antifeedant and larvicidal effects of 12 simple phenols and nine phenolic acids against *Spodoptera littoralis* (Boisd.). Along the same lines, an acetone extract of *Malpighia emarginata* DC. bagasse, which contained many phenolic compounds such as gallic acid, epigallocatechin gallate, catechin, *p*-coumaric acid, salicylic acid, and quercetin, prolonged the pre-pupal stage and increased the mortality of caterpillars of fall armyworm (*Spodoptera frugiperda* (J.E. Smith; Lepidoptera: Noctuidae)) [29]. Quick and cheap crop pest control solutions were introduced by Mkenda et al. [30] in Tanzania using aqueous leaf extracts of *Tephrosia vogelii*, *Tithonia diversifolia*, *Vernonia amygdalina* Delile, and *Lippia javanica* (Burm.f.) Spreng., which provided effective control against common pests of the bean-plant-like *Phaseolus vulgaris* L. and *Empoasca fabae*.

### 2.4. Survey and Population Density of Insects of P. vulgaris in a Field Treated with the SCG Extract

**Figure 4:**
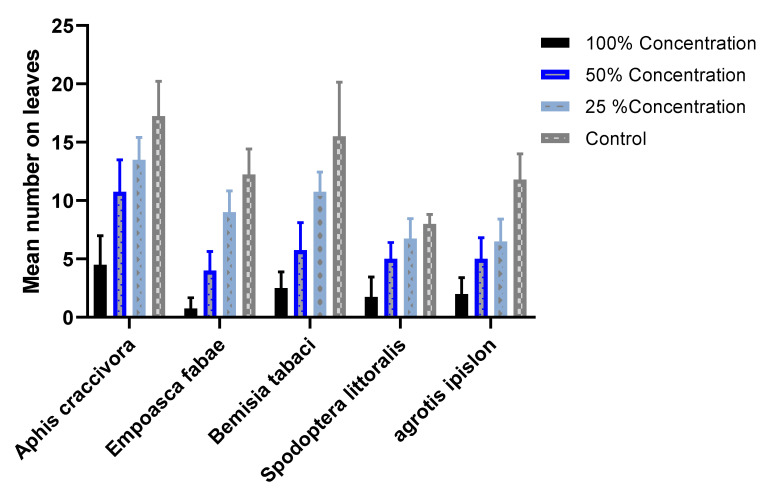
The mean numbers of insects collected from plots of each treatment.

The data presented in Figure 4 show the mean numbers of the main insects found in the field; aphid (*Aphis craccevora*) specimens numbered 4.5, 10.7, 13.5, and 17.3 at plants treated with 100, 50, and 25% of the SCG extract and the control, respectively. Statistical analysis showed extremely significant differences between the treatments and the control for the population density and among the various insects collected (*p*-values < 0.0001). Jassed (*Empoasca fabae*) was recorded as having 0.75, 4, 9, and 12.7 with treatments of 100, 50, and 25% of the SCG extract and the control, respectively. In the case of the whitefly, *Bemisia tabaci*, the numbers obtained were 2.5, 5.7, 10.7, and 15.5 with concentrations of the SCG extract of 100, 50, and 25% and the control (water). The number of cutworm larvae (*Agrotis ipsilon*) was counted during the earlier 35 days of the life of the plants because, after the third larval instar, those larvae shelter in soil, and it is challenging to find them on the foliage of plants. At the same time, it was possible to collect the larvae of cotton leafworms from the field during the entire season. Both of these two pests are responsible for damage to the foliage of this crop.

Data were recorded with the mean number of larvae caught in each plot according to the concentration; for *Spodoptera*, these numbers were 1.7, 5, 6.7, and 8.0 with 100, 50, and 25% of the SCG extract and the control, respectively. At an earlier plant age, the larvae of *Agrotis ipsilon* were collected from plants for each of the different treatments, and the mean numbers were 2, 5, 8.5, and 11.8 specimens/plant with the concentrations of 100, 50, and 25% of the SCG extract and the control, respectively. These results show that the high concentration of the 100% SCG extract provided a highly significant difference in comparison with the other lower concentrations of 50 and 25%, and much higher difference compared to the control.

**Figure 5:**
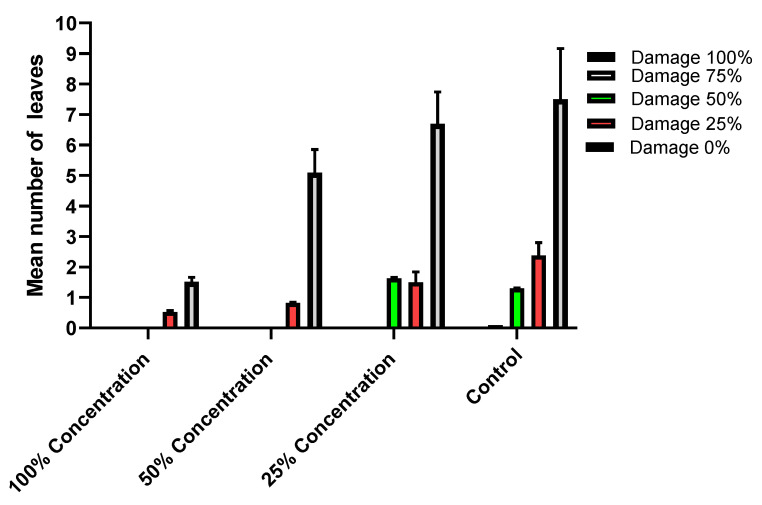
Effect of the SCG extract on the mean number of leaves damaged by chewing insects.

The decrease in the population numbers of *Spodoptera* and *Agrotis* larvae collected from the field was supported by the results, which were obtained twice per week. The mean number of leaves damaged by those insects was very low in the plots treated with the high concentrations of 100% and 50% of the SCG extract compared with the number in the plots with the lower concentration of 25% and the control (Figure 5). The statistical analysis showed more significant differences (*p*-value < 0.0001) between the treatments and control for both the damage percentage and the rate of damage, which supported the effects of different concentrations of the SCG extract on the feeding ability and, therefore, the damage to the plant leaves. These findings reinforce the assumption that the SCG extract acts as an antifeeding and repellent agent. The above results are in harmony with those of many previous works; for example, that of Pavela [28], who showed the antifeedant effect of phenolic extract on *Spodoptera littoralis* (Boisd.), that of Rahayu et al. [31], who studied the antifeedant activity of a leaf phenolic extract of two cultivars of *Carica papaya* L. on *Spodoptera -litura* F. larvae, and that of Tavaris et al. [32], who reviewed the antifeedant activity of many aqueous and ethanolic extracts from many plants, including *Ammi majus* L., *Apium graveolens* L., and *Melia azedarach* L., against *Agrotis ipsilon*. In the same context, liquid formulations of Neem Baan at 10 and 15 mL/L proved to be effective against *Bemisia tabaci* (90.7 to 93.3% in eggs, 93.3 to 97.1% in nymphs, and 92.4 to 94.2% reduction in whitefly adults after the third spray) [33]. 

### 2.5. Evaluation of Molecular Docking

An evaluation of the potential interaction between the predominant components of the methanolic SCG extract and the insecticide AChE enzyme (1QON) was conducted through a molecular docking study. The intermolecular interactions between the ligand and the target receptor were evaluated. The ideal pose was validated by aligning the X-ray bioactive conformer with the best-fitted pose of the same compound for the enzyme. The perfect pose of each molecule was selected according to the energy score, and the validation was considered satisfactory when the RMSD was smaller than 2.0 Å with respect to the crystallographic pose of the respective ligand [34].

**Figure 6:**
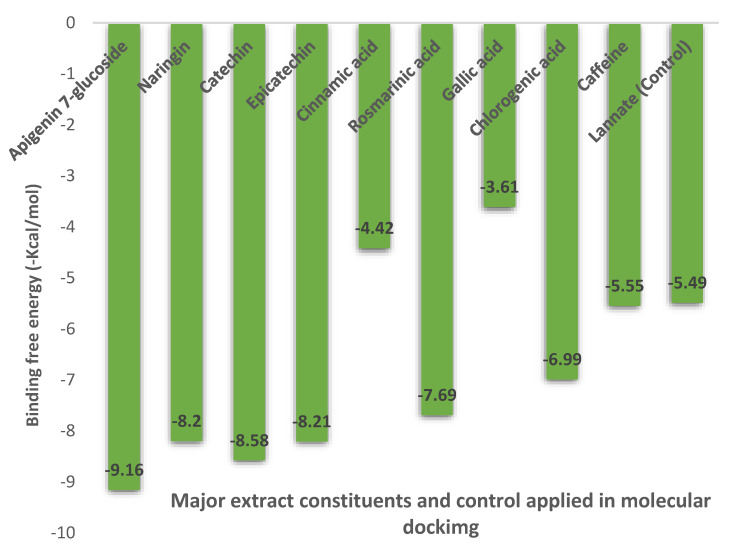
Binding free energy values were calculated through the molecular docking of the major constituents identified in the methanolic SCG extract and the AChE receptor.

The binding free energies (∆G) for the predominant components of the extract docked at AChE are shown in Figure 6, revealing the best poses obtained in the molecular docking analyses. The larger the peaks, the lower the ∆G and, consequently, the more significant the interaction between the receptor and the ligands with insecticidal abilities. Apigenin 7-glucoside displayed the best binding affinity compared to the other ligands and the control (lannate; −5.49 kcal/mol), with high docking scores (−9.16 kcal/mol), followed by catechin and epicatechin with −8.58 and −8.21 kcal/mol, respectively. Rosmarinic and chlorogenic acids showed median scores with −7.69 and −6.99 kcal/mol, while gallic acid had the lowest score (−3.61 kcal/mol) (Figure 6). The above results follow those of Tundis et al. [35] and Zengin et al. [36], where flavonoids, according to the former, and especially apigenin 7-glucoside, showed a potential anti-cholinesterase effect, while the latter showed an enzyme-inhibitory effect for the total methanolic extracts of both SCGs and coffee silverskin.

**Figure 7:**
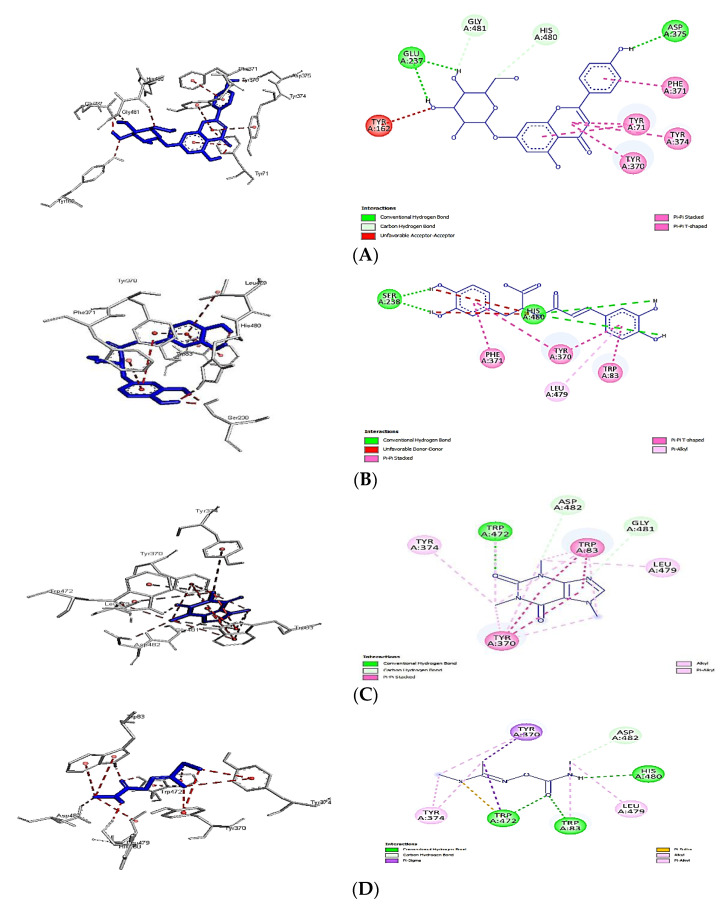
Interactions of (**A**) apigenin 7-glucoside, (**B**) rosmarinic acid, (**C**) caffeine, and (**D**) lannate (control) with the AChE receptor.

**Figure 8:**
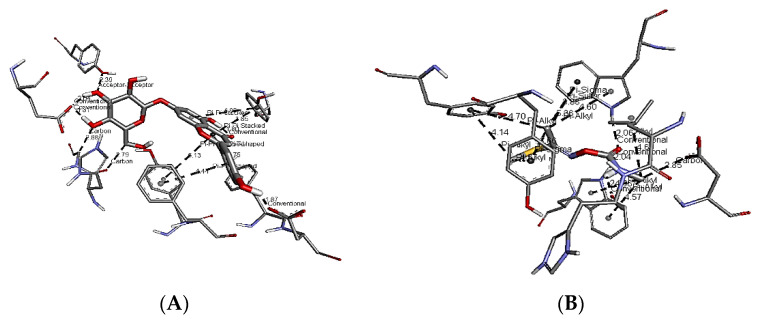
Types and distances of interactions between AChE and (**A**) apigenin 7-glucoside or (**B**) control (lannate).

Figure 7A–D show the interactions of apigenin 7-glucoside, rosmarinic acid, caffeine, and lannate (control) with the AChE receptor. These compounds represent the highest binding affinities among the different phenolics, flavonoids, and alkaloids identified in the SCG extract. The higher binding affinity of apigenin 7-glucoside was attributed to the crucial conventional hydrogen bonds formed with GLU237 and ASP375, the C-H interaction with GLY481 and HIS480, and, finally, the Pi–Pi interaction with TYR71, TYR370, and TYR374 (Figure 7A). The number of Pi–sigma interactions (Pi–alkyl), which primarily involved charge transfer, helped bind rosmarinic acid and caffeine firmly with the receptor residues LEU479, TYR370 TYR374, and TRP83. Again, similar Pi–Pi interactions with TYR370, TRP83, and PHE371 were shown for both, but caffeine had a unique conventional hydrogen bonding with TRP472 and carbon–hydrogen bonding with ASP482 and GLY481, while rosmarinic acid showed unparalleled hydrogen bonds with SER238 and HIS480. The types of bonds and their distances were the main reasons for the differences in binding affinity and free energy between each ligand and the control (Lannate), indicating the insecticidal ability of the tested molecules. The unique Pi–Pi interactions, C–H bonding, and distances of the bonds between ligands and enzyme moieties could make remarkable differences in binding affinity. For example, the distances of conventional H bonding between apigenin 7-glucoside and the AChE moieties were between 1.81 and 2.04 Å, shorter than the same between the control (lannate) and the receptor (2.04–2.24 Å). The same trend could be noted for the remaining bonds, as shown in Figure 8.

According to the literature, the molecular docking method used here identified a conformation that allowed the ligand to bind to the residues of the 1QON active sites, around the α-helix between the amino acid residues TYR370–TYR374, and around the β-sheet between the amino acid residues VAL478–HIS480. For the ligand, it was possible to see hydrogen bonds in common with residues TYR370 and HIS480. There was also a hydrophobic interaction with residues TYR71, TRP83, TYR370, PHE371, and LEU479 [37]. The interactions obtained after molecular docking of the compounds with the amino acid residues TRP83, TYR370, PHE371, and HIS480 of AChE were similar to those reported in the literature [38].

Generally, the main task of insecticidal agents, such as the potential compounds examined during the current study, is to irreversibly inhibit the production of the AChE enzyme, which is responsible for acetylcholine’s hydrolysis, which consequently terminates the nerve impulse through acetylcholine. The previous concept represents the initial mechanism for an extract or compound to be considered an insecticide for the larval phase [39]. Therefore, molecular docking represents an essential tool for observing the interactions formed inside the active site of AChE and the mechanism of elucidation of the biological action of the potential ligands applied.

## 3. Materials and Methods

### 3.1. Materials and Chemicals

SCGs were kindly supplied by Eng. Khaled El-Naggar of Misr Cafe (10th of Ramadan Ind. City, Cairo, Egypt). The material was dried using a hot-air oven at 38 ± 2 °C until reaching a 5% moisture content, and it was then stored for further extraction. Phenolic standards and methanol were purchased from Sigma (Sigma–Aldrich GmbH, Sternheim, Germany). All other chemicals used were of analytical grade and were obtained from either Sigma–Aldrich or Merck (Darmstadt, Germany).

### 3.2. Solvent Extraction Procedure

The extraction was carried out according to the optimized method by Mussatto et al. [8]. Extraction from the SCGs was performed with a 60% methanol concentration in a solvent/solid ratio of 40 mL/g of SCGs over 90 min at 60–65 °C in a water bath with magnetic agitation. Subsequently, the total content was centrifuged (2500× *g*, 4 °C, 20 min), and the supernatant (SCG extract) was filtered through 0.22 µm filters and stored at −20 °C in darkness until analysis or use. The volume of extract recovered was quantified and used for calculations.

### 3.3. Determination of Phenolic Acids and Flavonoids

The profiles and contents of phenolic acids and flavonoids in the methanolic SCG extract were analyzed by using high-performance liquid chromatography (HPLC) as described by Kim et al. [40]. A total of 2 µL of the extract was injected into the HPLC apparatus (Agilent 1100, G1329A ALS, Milford, MA, USA), which was equipped with a photodiode array detector (PDA 2998; Waters, Milford, MA, USA), quaternary pump, and autosampler. Separation was performed on a Symmetry C18 (4.6 mm × 150 mm, 3.5 μm) column (Waters, Milford, MA, USA) at 20 °C. The mobile phase was solvent A (2.5% acetic acid, *v*/*v*) and solvent B (acetonitrile). The following gradient was applied: 3–9% B (0–5 min), 9–16% B (5–15 min), and 16–36.4% B (15–33 min), followed by an isocratic run at 100% of B (5 min) and reconditioning of the column (3% of B, 10 min). The flow rate was 1.0 mL/min. The solvent mixture was degassed in an ultrasonic bath before being used as a mobile phase. The concentration of the phenolic acids was determined from standard curves made with known concentrations of each compound. Different wavelengths were used to retrieve peak areas at 280, 320, and 360 nm in order to maximize the generated signal and reduce the detection and quantitation limits.

### 3.4. Oviposition Deterrent Activities of the SCG Extract against Female Moths (Spodoptera littoralis)

Twelve plastic jars with a volume of 3 kg (15 cm in diameter and 30 cm high) were divided into four groups according to the applied concentrations (100, 50, 25%, and control), with three replicates. Distilled water was used for dilution and as a control. Ten pupae were chosen at the same age—five males and five females—and put inside each jar, and Nerium plants (*Nerium oleander*) were selected for oviposition. For adults that emerged from the pupae, three plants of *Nerium oleander* treated with each extract concentration were added inside the jars, which were covered with marshaling cloth and held tight with rubber bands. Adults were left until copulation and inspected daily to count how many batches of eggs had been laid on the plant per 5 females until all insects died.

### 3.5. Insecticidal Effect of the SCG Extract on the Percentage Mortality of Spodoptera littoralis

This test was performed using 16 Petri dishes (15 cm in diameter) divided into four groups for three concentrations of SCG extract (100, 50, and 25%) in addition to the control (water). Twelve leaves of the same size from the *P. vulgaris* plant were sprayed with the extract at each concentration. Five cotton leaf worms at the beginning of the fifth larval instar were added to each plate. The number of survivals in the treated dishes was inspected daily to record the percentage of mortality.

### 3.6. Survey and Population Density of Insects of P. vulgaris in the Field Treated with the SCG Extract

Field studies were performed in Bheira Governorate from the middle of February 2021 until the end of May 2021. The experimental area at the agricultural experiment station of the National Research Center, Nubaria region, Egypt (latitude 30.8667 N, and longitude 31.1667 E, and mean altitude of 21 m above sea level) was chosen, 140 km away from Cairo. The field was rectangular (17.5 m in width, 29 m in length), and the total area of this field was 507.5 m. The field was divided into two parts in the width of the land; each part was 8.4 m, separated by a 70 cm line. Within every two plots, on the side of the length, the barrier space was 50 cm. Each plot was 8.4 m in width and 3.20 m in length. Bronco cultivars of green bean seeds were provided by the Crop and Seed Propagation Department of the Agricultural Research Center of the Ministry of Agriculture (Giza, Egypt). Experimental and field studies were carried out following relevant institutional, national, and international guidelines and regulations. Two seeds were planted per hole, and they were thinned three weeks after seedling emergence. Manual weeding was performed, and herbicide was applied. The seeds of the green bean, *P. vulgaris*, were sown in plots in the middle of February and harvested in June. Surveying of the main key pests of *P. vulgaris* was done with different methods, i.e., yellow-colored sticky traps, sweep nets, and direct counting. The total numbers of each pest were recorded, and the mean numbers were calculated for the various concentrations and for every pest.

### 3.7. Molecular Docking

The crystal structure of AChE (PDB ID: 1QON) was obtained from the Protein Data Bank (PDB) (https://www.rcsb.org/, accessed on 6 October 2021), and it was prepared as a receptor by removing water and co-crystallized ligands and ions, then protonated using the Pymol software (Ver. 2.5.1) [41]. Meanwhile, the 3d structures of the ligands, which were downloaded from the PubChem database (http://pubchem.ncbi.nlm.nih.gov/, accessed on 6 October 2021), were optimized by using the MMFF94 force field by Avogadro Software (Ver. 1.2.0) [42]. According to the specified recommendations, the docking process was performed using combined validated AutoDock 1.5.6 tools with Vina [43]. Briefly, polar hydrogen atoms and Kollman charges were assigned for the receptor protein, while the addition of hydrogens and protonation with set torsions were automatically performed for the ligands. The grid box dimension was fixed at 40 Å, 40 Å, and 40 Å for the x, y, and z coordinates with a spacing of 0.375 Å, and the grid center (32.5, 68.3, and 11.2) was based on DeepSite, which is a binding pocket predictor that uses neural networks (https://playmolecule.com/deepsite/, accessed on 6 October 2021) [44]. A genetic algorithm was used to evaluate the parameters with the default setting, and the Lamarckian GA (4.2) was employed for the docking simulations. The conformers with the lowest Gibbs free binding energy (estimated as ∆G in kcal/mol) were selected for post-docking analysis. The Discovery Studio software (Ver. 21.1.0.20298) was used to visualize and analyze the interactions of the best-docked complexes.

### 3.8. Statistical Analysis

The results were expressed as mean values and standard deviations from at least three replicates. The statistical analysis of the data collected from the insecticidal activity was performed by using the Microsoft Excel program. By comparing one-way ANOVA and the least significant difference (*p* < 0.05) test, the significant differences among extract concentrations were assessed to differentiate the individual mean significant differences at the 0.05% level.

## 4. Conclusions

A deterrent assay, contact bioassay, and lethal concentration analysis were performed to reveal the repellent, antifeedant, and oviposition deterrent effects of a methanolic SCG extract against the main pests of green bean (*Phaseolus vulgaris*). The extract, which was rich in phenols and flavonoids, showed a promising insecticidal impact and oviposition deterrent efficiency based on the concentration of the extract applied compared to the control. A molecular docking study revealed that flavonoids and phenolic acids displayed higher activity through interactions with insecticidal AChE, which was in agreement with the laboratory and field studies. The above findings open prospects toward exploiting enormous amounts of agro-wastes, such as SCGs, as eco-friendly bioinsecticides and avoiding the global health and environmental problems of such food-industrial solid wastes.

## Figures and Tables

**Table 1:** Contents of phenolic acids, flavonoids, and caffeine determined in the methanol SCG extract.

		**Phenolic Acid Contents of the SCG Extract**			
**Compound**	**Rt (min.)**	**Quantities** **(µg/g) ^a^**	**Compound**	**Rt (min.)**	**Quantities** **(µg/g)**
Gallic acid	2.224	32.51 ± 2.08	Sinapic acid	20.523	10.1 ± 1.24
Protocatechuic acid	5.771	2.07 ± 0.74	(S)- (-)-Rosmarinic acid	20.534	0.53 ± 0.22
p-Hydroxybenzoic acid	8.609	4.37 ± 0.88	Ferulic acid	23.739	0.37 ± 0.14
Gentisic acid	9.351	0.26 ± 0.05	Salicylic acid	26.793	7.61 ± 1.05
Chlorogenic acid	12.500	8.74 ± 1.05	p-coumaric acid	30.072	0.16 ± 0.11
Caffeic acid	13.070	6.41 ± 0.74	Cinnamic acid	37.365	979.38 ± 4.78
Syringic acid	13.067	3.41 ± 0.41	(R)- (+)-Rosmarinic acid	37.363	163.1 ± 3.74
Vanillic acid	17.659	2.08 ± 0.56	-		-
		**Flavonoid Contents of the SCG Extract**			
**Compound**	**Rt (min.)**	**Quantities** **(µg/g)**	**Compound**	**Rt (min.)**	**Quantities** **(µg/g)**
Catechin	11.141	14.55 ± 1.47			
Epicatechin	13.494	10.08 ± 2.37	Apigenin-7-glucoside	36.828	1534.22 ± 7.74
Naringin	34.906	86.94 ± 3.15	Chrysin	53.646	1.01 ± 0.14
		**Alkaloid Contents of the SCG Extract**			
**Compound**			**Rt (min.)**	**(µg/g)**	
Caffeine			13.989	1322.2 ± 5.71	

^a^ Values represent averages *±* standard deviations for triplicate experiments.

## Data Availability

The data presented in this study are available in this article.
